# Multi-Omics Profiling of the Hepatopancreas of Ridgetail White Prawn *Exopalaemon carinicauda* Under Sulfate Stress

**DOI:** 10.3390/ijms27021056

**Published:** 2026-01-21

**Authors:** Ruixuan Wang, Chen Gu, Hui Li, Libao Wang, Ruijian Sun, Kuipeng Fu, Wenjun Shi, Xihe Wan

**Affiliations:** 1 National Demonstration Center for Experimental Fisheries Science Education, Shanghai Ocean University, Shanghai 201306, China; 18530040839@163.com (R.W.);; 2 Institute of Oceanology & Marine Fisheries, Nantong 226007, China

**Keywords:** sulfate stress, *Exopalaemon carinicauda*, hepatopancreas, physiology, transcriptomics, proteomics

## Abstract

With intensifying global climate change and human activities, and with regional topography interactions, soil and water salinization has intensified, posing major ecological and environmental challenges worldwide. Here, we integrated histology, transmission electron microscopy, RNA sequencing (RNA-seq) and data-independent acquisition (DIA)-based proteomics to profile hepatopancreas responses of *Exopalaemon carinicauda* during acute sulfate stress (≤48 h). Sulfate exposure disrupted tubular architecture and organelle integrity, consistent with early cellular injury. Multi-omics analyses revealed metabolic reprogramming marked by suppressed glycolysis (e.g., HK2, ENO) and enhanced oxidative phosphorylation (e.g., ATP5F1B), together with activation of calcium signaling (e.g., SLC8A1, ADCY9) and reinforcement of antioxidant/one-carbon and glucose-branch pathways (e.g., SHMT2, PGAM2). These coordinated transcript–protein changes indicate a shift from rapid cytosolic ATP supply to mitochondrial ATP production while buffering Ca^2+^ overload and reactive oxygen species. Collectively, our results delineate the physiological and molecular adjustments that enable *E. carinicauda* to cope with sulfate conditions and provide mechanistic targets for selective breeding and water-quality management in saline–alkaline aquaculture.

## 1. Introduction

With intensifying global climate change and human activities, and with regional topography interactions, soil and water salinization has intensified, becoming a major global ecological and environmental challenge [1]. Saline–alkaline soils and waters encompass ~46 million ha, widely distributed across the northwest, northeast, and northern regions, spanning 19 provinces, municipalities, and autonomous regions. These waters include chloride-, carbonate-, and sulfate-based types [2,3,4,5], and due to high salinity, alkalinity, and complex ionic composition, they can reduce biomass and alter community composition, thereby restricting organismal survival, growth, and reproduction [6,7]. In recent years, the use of saline waters in aquaculture has emerged as an important strategy to expand culture capacity and improve water-resource utilization [8]. Shrimp farming is one of the fastest-growing sectors of global aquaculture, with farmed shrimp production now exceeding 5 million tonnes annually and being dominated by intensive systems in Asia [9,10,11]. China is a major producer and consumer of farmed shrimp, and the rapid expansion and diversification of its shrimp industry are driving increasing demand for alternative water resources, including inland saline–alkaline waters [9,12]. *Exopalaemon carinicauda*, a member of the family Palaemonidae, is a major commercial mariculture species distributed along the Yellow and Bohai Sea coasts [13]. In addition to these biological traits, *E. carinicauda* has been successfully farmed in coastal and inland saline–alkaline ponds and is regarded as a promising candidate species for the efficient utilization of saline–alkaline water resources [14,15,16]. This species is considered an ideal experimental organism among crustaceans due to its transparent body, large egg size, high fecundity, and short reproductive cycle [17,18]. It is also recognized as one of the most important cultured species for developing saline water fisheries, owing to its broad salinity tolerance, rapid growth rate, and strong environmental adaptability [19,20]. According to aquaculture statistics, the farming area of *E. carinicauda* in China reached ~4000 ha in 2016, with an annual production of about 100,000 t, and a considerable proportion of this output now comes from saline–alkaline ponds in coastal cities such as Dongying, Shangdong province, and Cangzhou, Hebei province [14]. In these ponds, *E. carinicauda* is commercially reared at salinities of approximately 5–8‰ and carbonate alkalinities of about 1.4–13 mmol/L, highlighting its suitability for large-scale culture in saline–alkaline waters [16,20]. Therefore, *E. carinicauda* is both a key target species for saline–alkaline shrimp farming and a suitable model organism for investigating saline–alkaline adaptation mechanisms in crustaceans.

Existing studies have largely focused on fish and crustaceans in carbonate-type saline waters, where carbonate alkalinity is considered a major stressor affecting survival, growth, and reproduction in saline–alkaline environments [21,22,23]. For example, the impact of saline water on the antioxidant and phosphorylation capacities of *Gymnocypris przewalskii* has been investigated, and immune function in Pacific white shrimp *Litopenaeus vannamei* was found to be suppressed under carbonate stress [20,24]. Furthermore, a few studies have explored the molecular mechanisms of *E. carinicauda* in carbonate-type saline waters. For instance, research has examined the effects of this water on the antioxidant capacity of *E. carinicauda*, revealing preliminary changes in the expression of several relevant genes and proteins [14,25,26]. Sulfate-type saline water is one of the primary types in China and is widely distributed in inland areas of Northwest China, where it significantly affects agricultural production. Compared with carbonate-type saline water, sulfate-type waters are dominated by SO_4_^2−^ and Na^+^, and SO_4_^2−^ is more difficult to leach [27,28]. In sulfate-dominated saline–alkaline environments, increased osmotic pressure and enhanced ion toxicity often inhibit the growth and survival of aquatic organisms. Prolonged exposure to high concentrations of SO_4_^2−^ can even impair the physiological functions of aquatic animals, including antioxidant enzyme activity, tissue structure, and reproductive capacity, thereby reducing population survival and reproductive success [29,30]. Carbonate stress causes more direct disruption of ion and acid–base homeostasis, while sulfate stress shows a concentration- and time-dependent shift from compensatory regulation to decompensation and tissue damage [31,32,33,34,35]. As a result, SO_4_^2−^-dominated saline–alkaline conditions may impair growth and survival of aquatic organisms. Research on how sulfate-type saline–alkaline waters affect tissue injury and physiological function in *E. carinicauda* remains limited and lacks comprehensive, systematic analysis. This gap makes it difficult to provide sufficient theoretical support and technical guidance for the culture of *E. carinicauda* in saline waters.

This study aimed to evaluate tissue injury and related physiological indices in *E. carinicauda* exposed to sulfate-type saline–alkaline waters and to explore underlying physiological responses. Specifically, we assessed tissue damage by determining the median lethal concentration (LC_50_) at multiple time points and analyzed changes in physiological indicators under alkaline stress. Additionally, we integrated transcriptomic and proteomic techniques to investigate the molecular responses of *E. carinicauda* in sulfate-based saline–alkaline environments. Integrating transcriptomics with proteomics is necessary because mRNA expression does not always reflect protein abundance or activity; combining these two complementary layers of information enables a more comprehensive and functionally meaningful characterization of the stress-response network. Using multi-omics approaches, we sought to uncover adaptation strategies of *E. carinicauda* under high-salinity, alkaline conditions. This work provides scientific evidence and technical support for sustainable aquaculture of *E. carinicauda* in saline–alkaline waters and offers a reference for optimizing aquaculture models and promoting long-term development of saline–alkaline fisheries.

## 2. Results

### 2.1. The Variation in pH, Salinity, and Major Ions in Water Bodies with Different Sulfate Concentrations

The measured values of pH and salinity in water bodies with different sulfate concentrations are shown in Table S1. There is a significant difference in salinity among the water bodies with different sulfate concentrations (*p* < 0.05), but no significant difference in pH (*p* > 0.05), and both salinity and pH in this study are within the previously reported suitable ranges for the survival and growth of *E. carinicauda* (salinity 20–40‰; pH 4.5–10.1) [20,36]. The variation in major ions in water bodies with different sulfate concentrations is shown in Table S2. When the sulfate concentration in the water increases from 30 (control group) to 200 mmol/L, the contents of K^+^, Ca^2+^, Mg^2+^, Cl^−^, CO_3_^2−^, and HCO_3_^−^ do not show significant differences (*p* > 0.05), while the Na^+^ content significantly increases (*p* < 0.05).

### 2.2. Histomorphometric Analysis

**Figure 1:**
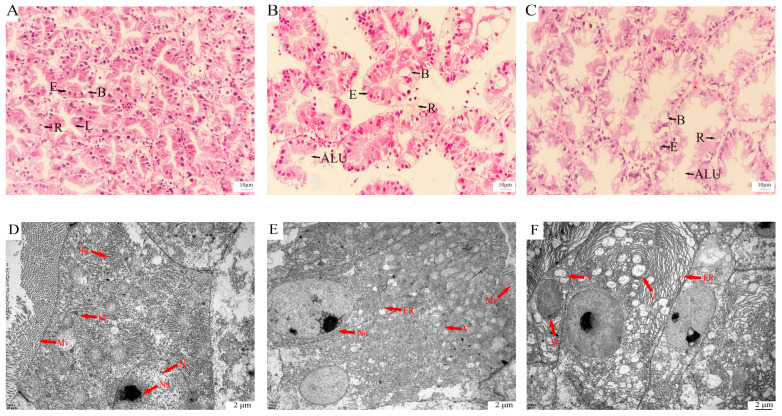
Histological and ultrastructural changes in the hepatopancreas of *Exopalaemon carinicauda* under sulfate stress. Panels (**A**–**C**) show HE-stained sections at 0, 24, and 48 h, respectively; panels (**D**–**F**) show TEM images of the hepatopancreas at 0, 24, and 48 h, respectively. B, B cells (Blasenzellen); R, R cells (Restzellen); E, E cells (Embryonalzellen); L, lumen; ALU, abnormal lumen; Mv, microvilli; M, mitochondria; N, nucleus; Nu, nucleolus; ER, endoplasmic reticulum; V, vacuolization.

H&E staining (Figure 1A–C) revealed pronounced hepatopancreatic remodeling in *E. carinicauda* under sulfate stress. In the control group, hepatopancreatic tubules were regularly arranged in a stellate pattern with uniform borders and abundant B and R cells (Figure 1A). After 24 h, tubules became irregular with an apparent increase in B cells, focal disruption of the basal lamina, and luminal dilation (Figure 1B), consistent with a rise in the lumen/empty-space area fraction from 22.9% (0 h) to 42.4% (24 h) and an increase in the upper-tail equivalent diameter of luminal profiles from 10.6 μm to 11.7 μm. By 48 h, tubule boundaries were blurred, luminal architecture was abnormal, and basement membranes of many tubules ruptured (Figure 1C); quantitatively, lumen expansion remained elevated (34.9% at 48 h), with further enlargement of luminal profiles (upper-tail equivalent diameter 13.3 μm) and a higher proportion of markedly dilated lumina (>~110 μm^2^; 6.9% at 0 h, 9.9% at 24 h, and 12.9% at 48 h).

TEM analysis (Figure 1D–F) showed progressive ultrastructural injury in the hepatopancreas of *E. carinicauda* under sulfate stress. In the control group, hepatic tubule cells were intact, with dense cytoplasm, clearly visible nucleus/nucleolus, neatly arranged microvilli, and abundant, well-organized endoplasmic reticulum; mitochondria displayed smooth outer membranes and regularly arranged cristae (Figure 1D). After 24 h, lesions became evident: microvilli loosened, the ER lost its characteristic organization, and focal ER swelling/rupture generated vesicle-like structures (Figure 1E). Quantitatively, electron-lucent vesiculation/vacuolization occupied ~4.09% of the cytoplasmic field at 0 h and remained similar at 24 h (~3.57%), while vesicle/vacuole profiles increased modestly (80 → 87 per field). By 48 h, severe lesions were observed, including swollen mitochondria with partial cristae loss and markedly swollen/fragmented ER with extensive rupture and abundant vesicle formation (Figure 1F); correspondingly, vesiculation/vacuolization rose sharply to ~9.60% and vesicle/vacuole counts surged to 177 per field.

### 2.3. Transcriptomic Analysis Under Sulfate Stress

Table S3 summarizes the RNA-seq results of the transcriptomic response of *E. carinicauda* under sulfate stress, and the data from different experimental groups of samples have been statistically analyzed for sequencing quality and base distribution. The table presents the initial raw data (RawData) for each sample, as well as the number and proportion of bases under different quality control criteria, including filtered data for Q20 and Q30. Additionally, the proportion of unknown bases (N%) that appeared during sequencing and the percentage of GC content for each sample were calculated. High-quality data analysis was ensured by the filtered data (CleanData). As shown in the table, the percentage of Q20 and Q30 filtered data for each sample is relatively high (all above 98%), indicating high data quality. Furthermore, the GC content varied little between samples, ranging from approximately 43.4% to 45.1%. The proportion of unknown bases remained between 0.05% and 0.07% for all samples. Transcriptome assembly yielded 68,832 transcripts/unigenes (GC = 39.45%; mean length = 1036 bp; N50 = 2005 bp; total assembled bases = 71,365,193 bp; longest = 35,431 bp; shortest = 201 bp).

**Figure 2:**
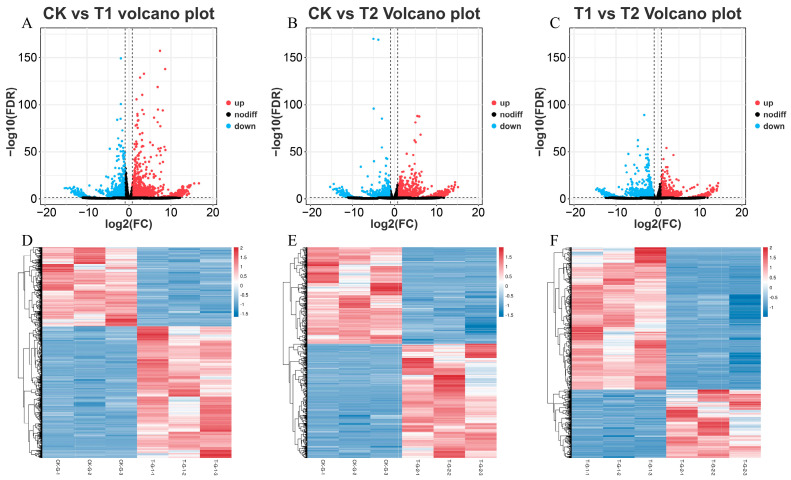
Comparison of DEG volcano plots and heatmaps. DEG volcano plots of CK vs. T1 (**A**), CK vs. T2 (**B**), and T1 vs. T2 (**C**), volcano plot showing upregulated genes (red), downregulated genes (blue), and non-significant genes (black) (the horizontal dotted line represents FDR < 0.05 and the vertical dotted lines represent an absolute fold change ≥ 2). DEG heatmaps of CK vs. T1 (**D**), CK vs. T2 (**E**), and T1 vs. T2 (**F**). Where each column represents a sample and each row a gene; red indicates higher expression levels, blue indicates lower expression.

To identify differentially expressed genes (DEGs) under sulfate treatment in *E. carinicauda*, comparative transcriptome analysis was conducted on samples from the CK, T1, and T2 groups (the criteria for significant differences were |log2FC| ≥ 1 and FDR < 0.05). The analysis identified a total of 4157 DEGs in the comparison between CK and T1, of which 2493 genes were upregulated, and 1664 genes were downregulated (Figure 2A). In the comparison between CK and T2, 2898 DEGs were identified, including 1409 up- and 1489 downregulated genes (Figure 2B). In the comparison between T1 and T2, a total of 3156 DEGs were detected, with 1053 up- and 2103 downregulated genes (Figure 2C). Furthermore, the heatmap analysis demonstrated that the expression profiles of DEGs were highly similar within each group, with the three replicates in each group clustering together distinctly from replicates in other groups.

**Figure 3:**
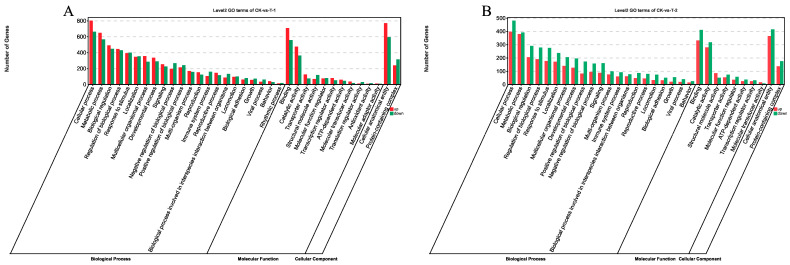
GO enrichment classification bar chart of DEGs. (**A**) GO pathway enrichment analysis of DEGs at 0 h and 24 h. (**B**) GO pathway enrichment analysis of DEGs at 0 h and 48 h.

To further examine regulatory patterns under different sulfate-stress levels, functional annotation of DEGs was performed. GO enrichment results for DEGs are shown in Figure 3. DEGs in T1 and T2 vs. CK were enriched across the three GO domains—biological process (BP), molecular function (MF), and cellular component (CC)—with significant terms defined by FDR < 0.05. Functional enrichment analysis indicated a significant enrichment of the differentially expressed genes (DEGs) from the comparison between CK and T1 in fundamental biological processes, such as cellular process, metabolic process, biological regulation, and single-organism process. The enriched cellular components primarily included cell, cell part, and organelles and membranes. In terms of molecular functions, the enrichment was mainly concentrated in binding activity and catalytic activity (Figure 3A). For the comparison between CK and T2, the biological process category showed the highest number of genes involved in cellular process, metabolic process, biological regulation, response to stimulus, and localization. Molecular function enrichment involved binding, catalytic activity, structural molecule activity, and transporter activity, while cellular component enrichment involved protein-containing complex and organelle (Figure 3B).

**Figure 4:**
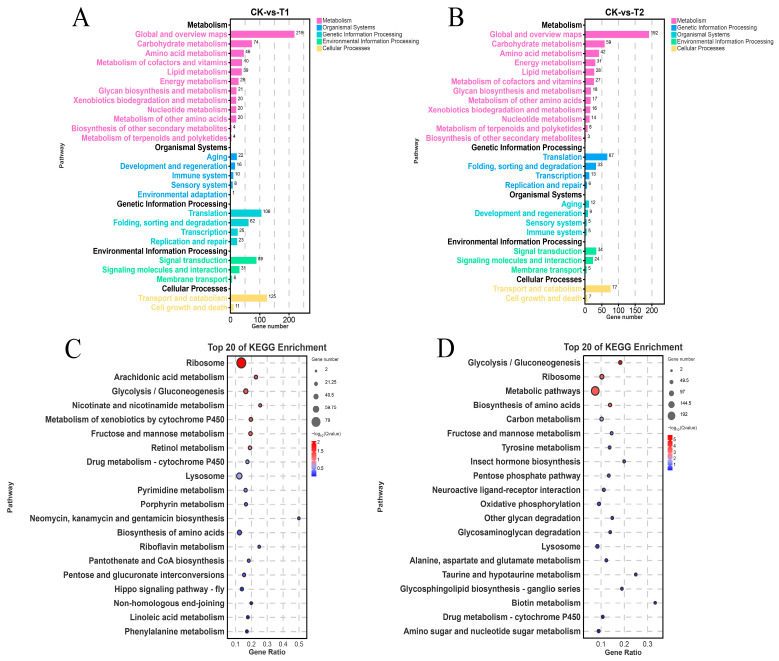
KEGG pathway classification and analysis of differentially expressed genes (DEGs). (**A**) CK vs. T1 group. (**B**) CK vs. T2 group. (**C**) Top 20 KEGG enrichment bubble plot for CK vs. T1. (**D**) Top 20 KEGG enrichment bubble plot for CK vs. T2.

We conducted a comparative analysis of KEGG pathways to identify the enrichment pathways of DEGs. The differentially expressed genes were categorized into five functional classes, including metabolism, genetic information processing, organismal systems, environmental information processing, and cellular process (Figure 4A,B). With FDR < 0.05, 133 pathways were enriched in CK vs. T1; the top 15 included ribosome, arachidonic acid metabolism, glycolysis/gluconeogenesis, nicotinate and nicotinamide metabolism, metabolism of xenobiotics by cytochrome p450, fructose and mannose metabolism, retinol metabolism, drug metabolism—cytochrome p450, lysosome, pyrimidine metabolism, porphyrin metabolism, aminoglycoside (neomycin/kanamycin/gentamicin) biosynthesis, biosynthesis of amino acids, riboflavin metabolism, and pantothenate and CoA biosynthesis; these relate mainly to energy metabolism, amino-acid biosynthesis, and lipid metabolism (Figure 4C). For CK vs. T2, 122 pathways were enriched; the top 15 included glycolysis/gluconeogenesis, ribosome, metabolic pathways, biosynthesis of amino acids, carbon metabolism, fructose and mannose metabolism, tyrosine metabolism, insect hormone biosynthesis, pentose phosphate pathway, neuroactive ligand–receptor interaction, oxidative phosphorylation, other glycan degradation, glycosaminoglycan degradation, lysosome, and alanine/aspartate/glutamate metabolism, reflecting enrichment in core metabolic and biosynthetic pathways (Figure 4D).

### 2.4. Proteomic Analysis Under Sulfate Stress

**Figure 5:**
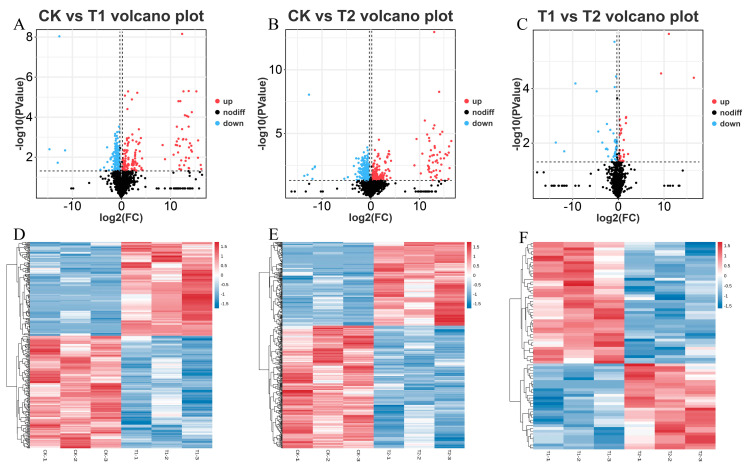
Comparison of DEP volcano plots and heatmaps. (**A**) Volcano plot of protein abundance quantification in the hepatopancreas of the CK group compared to the T2 group. (**B**) Volcano plot of protein abundance quantification in the hepatopancreas of the CK group compared to the T1 group. (**C**) Volcano plot of protein abundance quantification in the hepatopancreas of the T2 group compared to the T2 group. Red dots represent upregulated proteins, blue dots represent downregulated proteins, and black dots represent unchanged proteins. (the horizontal dotted line represents *p* < 0.05 and the vertical dotted lines represent an absolute fold change ≥ 2). DEP heatmaps for CK vs. T1 (**D**), CK vs. T2 (**E**), and T1 vs. T2 (**F**). Where each column represents a sample and each row a protein; red indicates higher expression levels, blue indicates lower expression.

In this study, a total of 15,723 identified spectra and 14,018 peptides were obtained, which were mapped to 2240 proteins. The MS/MS spectra were searched against a UniProt-based protein sequence database, and the identified proteins were functionally annotated using the GO, KEGG, and KOG databases. Among the 2240 proteins, 2010 (89.7%) were assigned GO terms, 1156 (51.6%) were mapped to KEGG pathways, and 1787 (79.8%) had KOG annotations; in total, 2094 proteins (93.5%) were annotated in at least one database, whereas only 146 proteins (6.5%) remained unannotated. A total of 635 differentially expressed proteins (DEPs) were identified in the hepatopancreas of *E. carinicauda* (|log2FC| ≥ 1, *p* < 0.05). In the comparison between CK and T1 (Figure 5A), 336 DEPs were identified, including 153 up- and 183 downregulated proteins. In the comparison between CK and T2 (Figure 5B), 478 DEPs were identified, with 194 up- and 284 downregulated proteins. In the comparison between T1 and T2 (Figure 5C), 75 DEPs were identified, consisting of 31 up- and 44 downregulated proteins. Furthermore, the heatmap demonstrated that the expression patterns of DEPs were highly similar within each group, with the three replicates in each group clustering closely together and distinctly separating from replicates in other groups.

**Figure 6:**
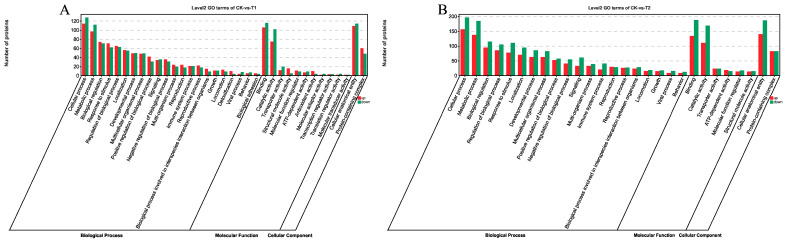
GO enrichment classification bar chart of DEPs. (**A**) GO pathway enrichment analysis of DEPs at 0 h and 24 h. (**B**) GO pathway enrichment analysis of DEPs at 0 h and 48 h.

According to GO annotation, the DEPs between the CK and T1 groups were classified into 35 distinct categories (Figure 6A), while the DEPs between the CK and T2 groups were classified into 39 distinct categories (Figure 6B), spanning biological process (BP), molecular function (MF), and cellular component (CC). As shown in the figure, GO enrichment analysis of DEPs revealed that the most significant BP term following acute sulfate stress in the CK and T1 groups was cellular process (GO:0009987), followed by metabolic process (GO:0008152) and biological regulation (GO:0065007). For MF, the top three significantly enriched terms were binding (GO:0005488), catalytic activity (GO:0003824), and transporter activity (GO:0005215). CC terms included cellular anatomical entity (GO:0110165) and protein-containing complex (GO:0032991). Similarly, in the CK and T2 groups, GO terms were primarily enriched in cellular process (GO:0009987), metabolic process (GO:0008152), binding (GO:0005488), catalytic activity (GO:0003824), cellular anatomical entity (GO:0110165), and protein-containing complex (GO:0032991).

**Figure 7:**
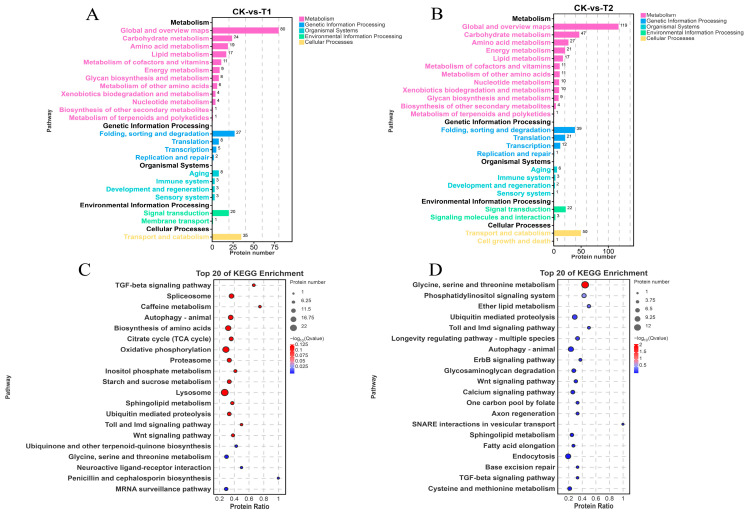
KEGG pathway classification and analysis of differentially expressed proteins (DEPs). (**A**) CK vs. T1 group. (**B**) CK vs. T2 group. (**C**) Top 20 KEGG enrichment bubble plot for CK vs. T1. (**D**) Top 20 KEGG enrichment bubble plot for CK vs. T2.

KEGG enrichment grouped DEPs into six classes (Figure 7A,B). Secondary category distributions were consistent with the transcriptome. In T1 vs. CK (Figure 7C), the top enriched pathways were glycine, serine, and threonine metabolism (ko00260); phosphatidylinositol signaling system (ko04070); and ether lipid metabolism (ko00565). In the comparison between T2 and CK (Figure 7D), the most significantly enriched pathway was the transforming growth factor-beta (TGF-β) signaling pathway (ko04350), followed by spliceosome (ko03040) and caffeine metabolism (ko00232). Specifically, three pathways were shared between T1/CK and T2/CK: TGF-β signaling (ko04350), glycine/serine/threonine metabolism (ko00260), and Toll and Imd signaling (ko04624).

### 2.5. Integrated Transcriptome–Proteome Analysis

**Figure 8:**
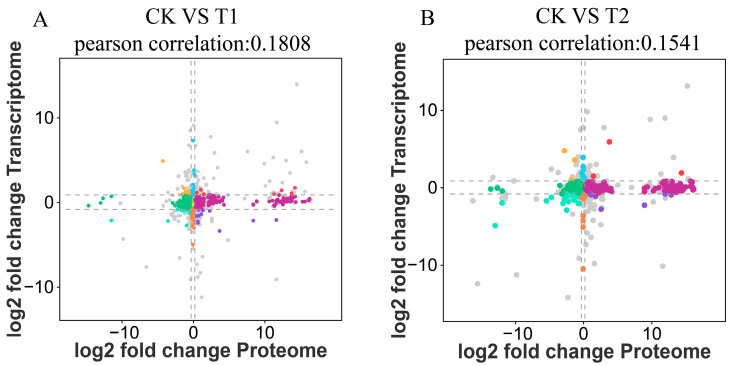
The relationship between transcriptomic and proteomic changes. (**A**) CK vs. T1, (**B**) CK vs. T2. Each dot represents a gene/protein, and different colors indicate distinct expression trends of genes between the two histological types. Grey dots denote genes that are not significantly differentially expressed in either the transcriptome or the proteome (genes: |log2FC| ≥ 1 and FDR < 0.05; proteins: |log2FC| ≥ 1 and *p* < 0.05).

To evaluate the relationship between transcriptomic and proteomic changes, we generated nine-quadrant plots comparing the log2 fold changes in mRNAs and their corresponding proteins (Figure 8). In both comparisons (CK vs. T1 and CK vs. T2), transcript and protein abundance changes were only weakly correlated (Pearson’s r = 0.1808 and 0.1541, respectively), with most features clustering around the origin; a subset of genes/proteins showed concordant up- or downregulation in both datasets.

**Figure 9:**
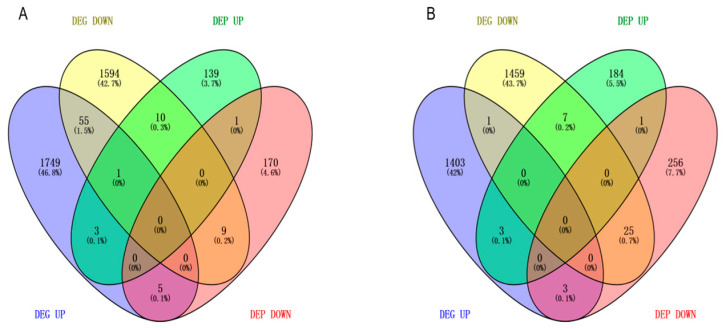
Differentially expressed genes (DEGs) and differentially expressed proteins (DEPs) distribution in transcriptome and proteome association analysis. (**A**) Wayne diagram between 0 h and 24 h correlation data. (**B**) Wayne diagram between 0 h and 48 h correlation data.

To further pinpoint hub genes and proteins, we assessed the correlation between transcriptomic and proteomic abundances, offering an integrated perspective on their relationship under sulfate-stress conditions. Consistency and differential expression analyses of the transcriptome and proteome was performed to investigate the relationship between DEGs and DEPs under sulfate stress in *E. carinicauda*. In the comparisons between 0 and 24 h and between 0 and 48 h, 23 and 35 significantly regulated matching DEGs/DEPs were identified, respectively, indicating a moderate overall correlation between the expression of hepatopancreatic proteins and mRNA (Figure 9). At 0 vs. 24 h, 3 DEGs/DEPs were consistently upregulated, 9 DEGs/DEPs were consistently downregulated, and 11 DEGs/DEPs showed opposite expression patterns (Figure 9A). At 0 vs. 48 h, 3 DEGs/DEPs were upregulated, 25 DEGs/DEPs were downregulated, and 7 DEGs/DEPs showed opposite expression patterns (Figure 9B).

**Figure 10:**
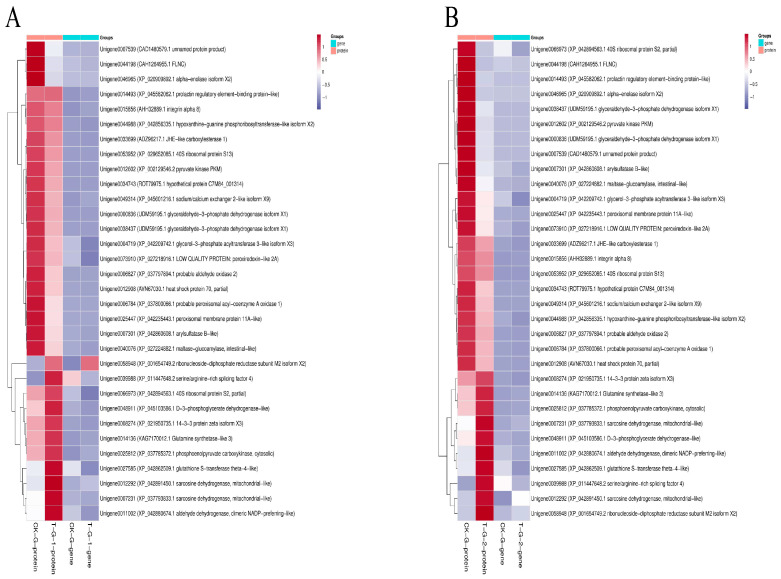
Significantly enriched differentially expressed genes (DEGs)/differentially expressed proteins (DEPs) in both transcriptome and proteome. (**A**) Hierarchical cluster analysis of 32 DEGs in 0 vs. 24 h group. (**B**) Hierarchical cluster analysis of 32 DEGs in 0 vs. 48 h group.

The DEGs/DEPs that were consistently upregulated between 0 and 24 h mainly include RRM2, GS2, and PCK2, while the DEGs/DEPs that were consistently downregulated between 0 and 24 h are primarily associated with metabolism, particularly “glycolytic metabolism,” “lipid metabolism,” and “fatty acid metabolism” (Figure 10A). The DEGs/DEPs that were consistently upregulated between 0 and 48 h are mainly involved in signal transduction pathways (GS2), energy metabolism (PCK2), and amino acid metabolism (Sardh), while those that were consistently downregulated between 0 and 48 h include glycolysis (GAPDH, PKM, ENO1), fatty acid metabolism (GPAT3, ACOX1), hormone and purine metabolism (HPRT), and oxidative stress and antioxidation (HSP70, PRDX) (Figure 10B).

**Figure 11:**
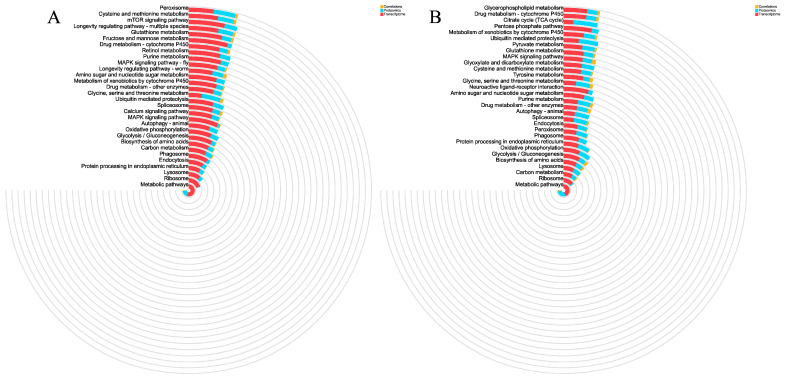
Integrated KEGG pathway analysis and concordance between transcriptomic and proteomic responses in *E. carinicauda* under sulfate stress. (**A**) Integrated KEGG pathway analysis for CK vs. T1, (**B**) Integrated KEGG pathway analysis for CK vs. T2.

Integrated KEGG pathway analysis of the combined transcriptomic and proteomic datasets further identified several pathways enriched at both the mRNA and protein levels (Figure 11), including metabolic pathways, ribosome, carbon metabolism, lysosome, biosynthesis of amino acids, glycolysis/gluconeogenesis, oxidative phosphorylation, protein processing in the endoplasmic reticulum, phagosome, peroxisome, endocytosis, spliceosome, autophagy–animal, drug metabolism–other enzymes, purine metabolism, and amino sugar and nucleotide sugar metabolism.

### 2.6. Quantitative Real-Time PCR (RT-qPCR) Validation

**Figure 12:**
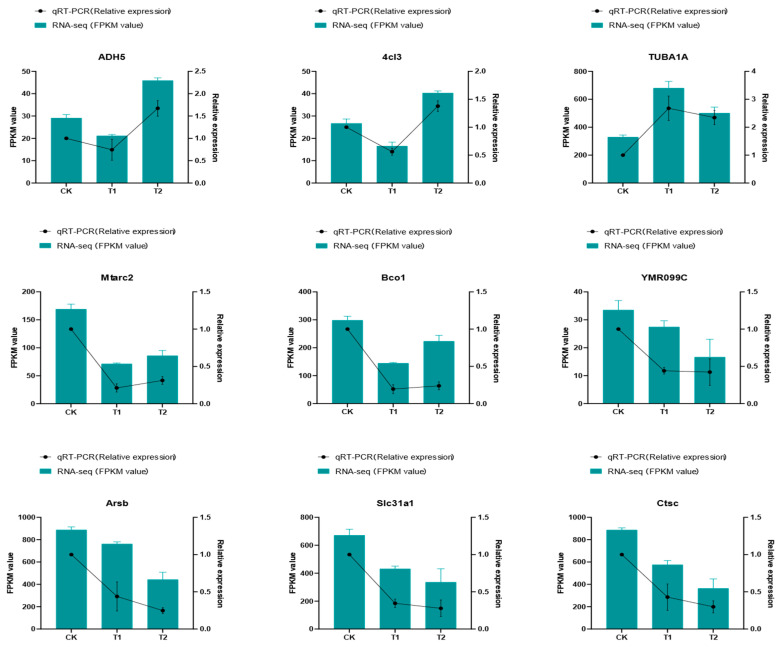
Expression of DEGs in three different stages.

To verify the reproducibility and accuracy of the RNA-seq data, RT-qPCR was used to quantify nine randomly selected DEGs. As shown in Figure 12, quantitative PCR (qPCR) validation of the nine selected differentially expressed genes (DEGs) showed expression trends that agreed with the transcriptomic data.

## 3. Discussion

**Figure 13:**
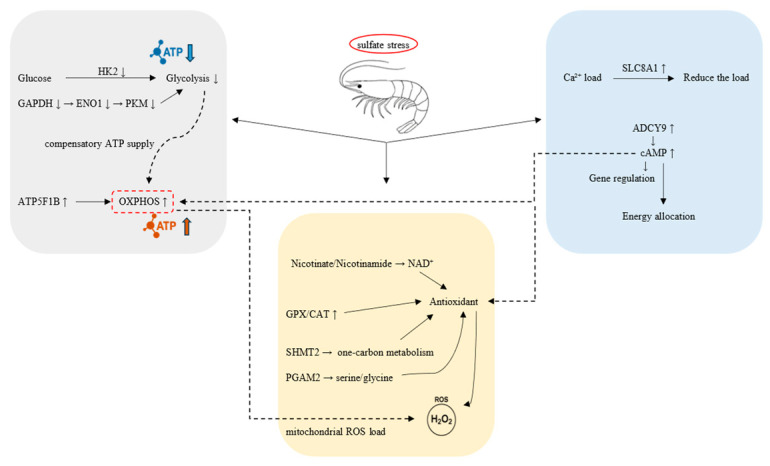
Sulfate stress-induced metabolic reprogramming and adaptive regulation in *E. carinicauda*.

Salinity significantly influences the growth and survival of aquatic organisms, impacting their disease resistance, feeding behavior, and distribution [37]. Although the metabolomic responses of *E. carinicauda* in carbonate environments have been investigated, there is a lack of studies on tissue damage and histological responses to sulfate stress [14]. Previous multi-omics studies under carbonate alkalinity in *E. carinicauda* have mainly highlighted gill- and hepatopancreas-based ion-transport and endocrine responses, including activation of carbonic anhydrase, Na^+^/H^+^ exchangers, aquaporins and adenosine triphosphate (ATP-binding) proteins, together with disorganization and vacuolization of gill and hepatopancreatic tissues and broad immune activation under high carbonate alkalinity [14,25]. By contrast, the sulfate regime used here (106 mmol/L SO_4_^2−^ at near-ambient pH) elicits a distinct hepatopancreas-centered pattern characterized by severe cavitation and loss of luminal structure, basement-membrane rupture, and pronounced mitochondrial and endoplasmic-reticulum lesions, accompanied by cross-omics evidence of glycolytic suppression, reinforced OXPHOS, activation of nicotinamide adenine dinucleotide, oxidized form (NAD^+^)/one-carbon-linked antioxidant metabolism, and upregulation of calcium ion (Ca^2+^)/cyclic adenosine monophosphate (cAMP) signaling. These differences suggest that, within saline–alkaline systems, carbonate stress primarily drives an “ion-transport/endocrine” mode of adaptation, whereas sulfate stress preferentially targets hepatopancreatic metabolic–organelle homeostasis and redox/ionic balance in *E. carinicauda*. Additionally, the complexity of gene regulation may lead to less accurate and comprehensive results in transcriptome analyses [38]. Distinct histological approaches emphasize different aspects of the stress response and can yield varying results. Consequently, a single histological dataset is unlikely to capture the full diversity of biomolecular responses under stress [39]. Consistent with this systems view, shrimp subjected to chronic pH stress exhibit growth inhibition, declines in immune and antioxidant capacity, and functional dysbiosis of the gut microbiota, indicating that chronic chemical stress causes coordinated, multi-pathway damage at the organismal level [40]. Therefore, the integration of multi-omics data (physiology, transcriptomics, proteomics) is essential to elucidate the functional mechanisms, regulatory pathways, and candidate genes involved in the biological process. The hepatopancreas is an important detoxification organ in crustaceans [41]. In our study, we observed cavitation and loss of luminal structure in the hepatopancreas after exposure to sulfate stress; we further propose that the up- or downregulation of certain genes under these conditions could activate or inhibit relevant metabolic pathways and, in turn, alter metabolite levels. Additionally, changes in metabolites can regulate gene expression through feedback mechanisms. In this study, we analyzed the effects of sulfate stress on the hepatopancreas of *E. carinicauda* in terms of physiological, transcriptomic, and proteomic responses. This complex adaptation is schematically summarized in Figure 13.

### 3.1. Energy Metabolic Changes Induced by Sulfate Stress

Sulfate stress affects the energy metabolism of aquatic animals. As a central ATP-yielding pathway, glycolysis scales with energy demand during salinity challenges [42]. Similarly, acute high-alkalinity exposure induces hepatopancreatic injury with oxidative stress and coordinated shifts in energy- and immunity-related pathways [43]—hallmarks of early metabolic reprogramming. Within this framework, hexokinase and enolase act as glycolytic gatekeepers, with hexokinase 2 (*HK2*) catalyzing the initial phosphorylation of glucose that channels carbon into downstream intermediates to sustain high metabolic output [44]. In our study, *HK2* expression was significantly downregulated under sulfate stress, suggesting a deliberate throttling of glycolytic flux and ATP production. Suppression of glycolysis has also been observed under a variety of stressors and in different taxa: in silver crucian carp, repression of *HK2* limits muscular glucose utilization and preserves glucose reserves [45]; in *Penaeus vannamei*, *HK* downregulation during high-fat adaptation reduces glucose catabolism and dampens glycolysis [46]; and under heat stress, *P. vannamei* similarly suppresses glycolysis to curb ATP consumption and reduce cellular burden [47]. Taken together, *E. carinicauda* undergoing sulfate stress appears to transiently downshift glycolysis as a protective, energy-conserving program while redirecting carbon toward auxiliary routes, such as fructose–mannose metabolism (upregulation of *Hex-t2*, *ALDOB*, and *ALDP*) and riboflavin metabolism (upregulation of *ENPP1* and *ENPP3*), to maintain metabolic balance and essential physiological functions during prolonged exposure.

At the protein level, sulfate stress induced a mitochondria-centered adjustment of the oxidative phosphorylation (*OXPHOS*) machinery: multiple OXPHOS genes were upregulated, with a prominent increase in the ATP synthase β-subunit ATP synthase F1 subunit beta (*ATP5F1B*). Building on previous work showing that mitochondria orchestrate cellular adaptation under sustained stress in crustaceans [48], we hypothesize that the upregulation of *ATP5F1B* is closely linked to the regulation of mitochondrial function. *ATP5F1B* encodes a core subunit of mitochondrial ATP synthase and plays a key role in ATP production via oxidative phosphorylation (OXPHOS) [49,50]. Consistent with a compensatory shift when glycolysis is constrained, cells can pivot to OXPHOS to maintain ATP; upregulated *ATP5F1B* is known to sustain ATP output under high-demand states or glycolytic limitation [51]. OXPHOS is closely related to innate immunity and the extent of the immune response [52]; enhanced OXPHOS capacity has also been linked to damage repair, immune maintenance, and antioxidant competence [53]. Accordingly, *ATP5F1B* upregulation appears to constitute a mitochondria-centered adaptation that elevates OXPHOS capacity; under glycolytic limitation, it can maintain ATP supply and mitigate the metabolic burden imposed by glycolytic inhibition.

Overall, *E. carinicauda* exposed to sulfate stress in this experiment exhibited a coordinated metabolic reprogramming that suppressed cytosolic glycolysis while reinforcing mitochondrial ATP production. At the transcriptional level, *HK2*, a key kinase regulating glucose entry into glycolysis, was significantly suppressed, indicating a downregulation of glycolytic flux and ATP production. This is concordant at the protein level with the decrease in core glycolytic enzymes (e.g., glyceraldehyde-3-phosphate dehydrogenase (*GAPDH*), alpha-enolase (*ENO1*), and pyruvate kinase M (*PKM*) in our dataset), together pointing to a system-level brake on rapid glucose catabolism. In parallel, both layers converge on a mitochondrial countermeasure: OXPHOS genes are upregulated and the ATP synthase β-subunit *ATP5F1B* increases at the protein level, indicating a mitochondria-centered compensation that sustains ATP output when glycolysis is constrained. In line with this, the ultrastructural data show swollen mitochondria with loss of cristae in hepatopancreatic cells after 48 h of sulfate exposure (Figure 1F), indicating that mitochondria are not only the main ATP providers but also primary targets of sustained stress. Thus, the shift from glycolytic throttling to enhanced OXPHOS likely reflects an attempt to preserve ATP supply for ion transport and tissue repair during progressive hepatopancreatic injury, as evidenced by the distortion and basement-membrane disruption of hepatopancreatic tubules (Figure 1B,C). A cross-layer concordance of downregulated glycolysis with reinforced OXPHOS indicates that post-transcriptional mechanisms sustain mitochondrial capacity against the backdrop of transcript-level energy conservation. Together, these cross-omics signals delineate a purposeful shift from glycolytic throttling to compensatory OXPHOS, a program that conserves resources, preserves ATP supply, and integrates immunometabolic support to enhance tolerance to sulfate stress.

### 3.2. Antioxidant and Metabolic Defense Responses Induced by Sulfate Stress

Under sulfate stress, redox imbalance emerges as a primary phenotype [54,55]. Prior evidence shows that niacin/nicotinamide metabolism buffers oxidative stress by sustaining the intracellular NAD^+^ pool and can cooperate with canonical antioxidant pathways [56]. From this, we hypothesize a two-arm defense in shrimp: niacin-driven NAD^+^ metabolism sustains enzymatic antioxidant capacity, and a tyrosine-derived product provides direct radical-quenching capacity. In our dataset, the hepatopancreas exhibited enrichment of tyrosine metabolism, consistent with mobilization of tyrosine-derived metabolites to reinforce intracellular antioxidant defenses. Tyrosine-related redox chemistry interfaces with enzymatic (e.g., glutathione peroxidase (GPX), catalase (CAT)) and non-enzymatic glutathione (GSH) systems [57]. This closes the pathway loop with the niacin side: niacin and nicotinamide maintain NAD^+^, which in turn supports antioxidant gene expression and enzyme function (e.g., GPX, CAT) during acute stress [57]. Antioxidant enzymes (e.g., peroxidase (POD), CAT) typically surge early and then decline with prolonged exposure, consistent with enzyme fatigue when reactive oxygen species (ROS) exceeds system capacity [58]. We therefore propose that *E. carinicauda* mounts an early NAD^+^-supported and tyrosine-driven antioxidant response, but, under sustained sulfate stress, must increasingly reallocate to these (e.g., nicotinic acid and tyrosine metabolism)—and potentially additional—routes to mitigate damage and preserve essential physiological functions.

Under sulfate stress, the antioxidant and metabolic defense of *E. carinicauda* showed significant regulation, notably the upregulation of serine hydroxymethyltransferase 2 (SHMT2) and phosphoglycerate mutase 2 (PGAM2), which are linked to one-carbon and glycolytic metabolism. This further demonstrates that cells respond to oxidative stress and maintain physiological homeostasis by mobilizing various metabolic pathways. Anchored in prior work, SHMT2 operates at a key entry point of one-carbon metabolism, supplying intermediates for nucleotide biosynthesis and repair and thereby stabilizing cellular function under oxidative load [59]. Elevated SHMT2 in *E. carinicauda* likely sustains DNA synthesis/repair during prolonged sulfate exposure, supporting stress adaptation through the one-carbon network. PGAM2 can influence serine/glycine-linked pathways and redox balance. In grouper, PGAM2 downregulation suppresses serine–glycine synthesis, implicating PGAM2 in oxidative-stress responses; thus, its upregulation here plausibly reinforces metabolic flexibility that buffers oxidative damage [60]. Converging with these metabolic adjustments, antioxidant enzymes—including GPX and CAT—were also elevated. Enhanced mitochondrial oxidative phosphorylation can increase GPX and CAT activities, thereby accelerating the clearance of H_2_O_2_ and related ROS, limiting macromolecular injury and preserving cellular homeostasis [61,62]. Collectively, these data indicate a coordinated adaptation in which sulfate stress is accompanied by SHMT2- and PGAM2-mediated metabolic support together with increased GPX/CAT capacity for peroxide detoxification, jointly stabilizing redox homeostasis and preserving cellular integrity.

Under sulfate stress, *E. carinicauda* exhibited a coordinated response from transcriptional regulation to protein functionality: by upregulating antioxidant enzymes and restructuring metabolic networks, it enhances peroxide clearance and stabilizes intracellular redox balance. Transcriptomic analysis reveals that the NAD^+^ and tyrosine metabolism pathways are enhanced, providing essential coenzymes and substrates to support the antioxidant enzyme system. Proteomic analysis shows that the enhancement in one-carbon metabolism mediated by SHMT2 and the regulation of glycolysis–serine diversion by PGAM2 ensure sufficient intracellular reducing power (nicotinamide adenine dinucleotide phosphate (NADPH)/GSH) for ROS clearance. These molecular adjustments align closely with the ultrastructural findings: at 24 h, early endoplasmic reticulum (ER) swelling and vesicle formation (Figure 1E) coincide with activation of antioxidant pathways, whereas by 48 h, pronounced mitochondrial swelling and loss of cristae together with extensive ER vacuolization (Figure 1F) indicate that ROS- and ER-stress-driven injury has overridden the protective capacity of these systems. These two pathways mutually support each other and work together, reflecting a multi-level coordination from gene expression to metabolic activity, ensuring the survival and homeostasis maintenance of the striped tail shrimp under sulfate stress.

### 3.3. Ionic Homeostasis and Signaling Regulation Responses Induced by Sulfate Stress

Under sulfate stress, ionic and signaling homeostasis is perturbed. Prior evidence establishes calcium signaling as a master regulator of ion balance and stress responses [63], and in crustaceans, chronic low salinity disrupts epithelia and induces ion-transport/osmoregulatory programs, underscoring the system’s plasticity under osmotic challenge [64]. Sulfate stress appears to impose an intracellular Ca^2+^ burden that requires rapid buffering and tight coupling to metabolic regulation, as evidenced by significant upregulation of calcium-signaling genes, including *SLC8A1* (NCX) and adenylate cyclase 9 (*ADCY9)*. *SLC8A1* provides a rapid calcium buffering mechanism to maintain ionic homeostasis, while elevated *ADCY9* would raise cAMP, a second messenger that tunes gene expression and energy partitioning to support the ATP-demanding work of ion transport—thereby coupling Ca^2+^ buffering to metabolic homeostasis. *SLC8A1* induction in *P. vannamei* enhances Ca^2+^ transport capacity under stress [65], and that cAMP centrally regulates growth and metabolic balance in aquatic vertebrates [66,67]. These results indicate that *E. carinicauda* maintains ionic homeostasis and enhances stress tolerance under sulfate exposure by coordinating Ca^2+^ and cAMP signaling—namely, coupling rapid NCX-mediated Ca^2+^ extrusion with *ADCY9*-driven cAMP signaling. At the tissue level, this signaling adjustment occurs in the context of marked structural damage of the hepatopancreatic epithelium, including dilation of tubule lumina, blurring of tubule boundaries, and focal disruption of the basal lamina (Figure 1B,C), as well as disorganization of apical microvilli (Figure 1E). The induction of *SLC8A1* and *ADCY9* therefore likely represents a compensatory mechanism that attempts to restore Ca^2+^ and osmotic balance and to preserve epithelial integrity in the face of sulfate-induced ionic disturbances that manifest morphologically as tubule distortion and barrier breakdown.

## 4. Materials and Methods

### 4.1. Sulfate Stress Experiments and Sampling

**Figure 14:**
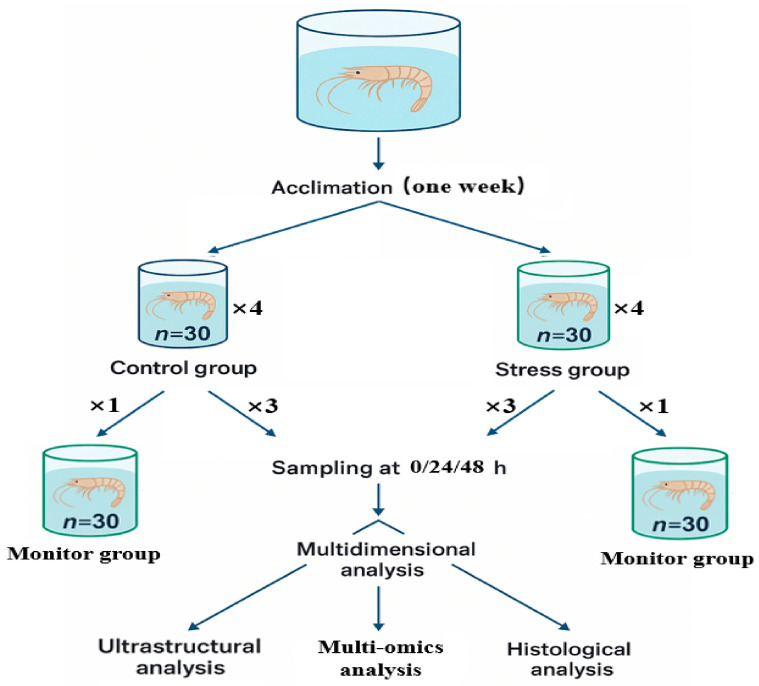
Experimental Design of *E. carinicauda* Under Stress Conditions.

The experimental *E. carinicauda* (mean length of 6.78 ± 0.31 cm; mean weight of 1.86 ± 0.26 g) were obtained from the Rudong Experimental Base of Marine Shrimp Culture, Jiangsu Marine Fisheries Research Institute. The sulfate exposure experiment was carried out from October to November 2023. The experimental water was natural seawater that was filtered, allowed to settle, and fully aerated (salinity: 25 ± 0.5 PSU; temperature: 24.0 ± 0.5 °C; pH: 8.0 ± 0.5). Based on pre-test results, the 48 h LC_50_ of sulfate for *E. carinicauda* was 99.9 mmol/L (95% CI: 55.229–106.643 mmol/L); thus, 106 mmol/L of SO_4_^2−^, close to the upper 95% CI limit of the 48 h LC_50_, was selected as the experimental sulfate concentration; pure seawater was used as the control group (SO_4_^2−^ = 30 mmol/L) (Tables S4 and S5). Seawater contains about 28 mmol L^−1^ of SO_4_^2−^, whereas sulfate concentrations in sulfate lakes can reach several hundred mmol/L. Thus, the experimental sulfate level used here (106 mmol/L of SO_4_^2−^) is higher than that of seawater but falls within the lower part of the natural range for sulfate-type saline–alkaline waters, representing a moderate sulfate enrichment rather than an extreme brine [68,69]. The experimental group was stabilized for 24 h. Four replicate tanks per treatment (*n* = 30 shrimp each) were set up, one tank was used for monitoring survival rate, cumulative mortality of *E. carinicauda* was recorded at three time points: CK (0 h), T1 (24 h), and T2 (48 h), with dead shrimp promptly removed (Table S6), other three tanks were used for taking samples. One-third of the water (pre-adjusted to the corresponding sulfate concentration) was replaced daily; all other husbandry conditions matched the acclimation period. Throughout the 48 h exposure period, temperature, dissolved oxygen and pH remained stable in all tanks, as shown in Table S7. At 0, 24, and 48 h, the hepatopancreas of shrimp from each tank was collected for histological evaluation, electron microscopy, and frozen section analysis. For each time point, live shrimp were first anesthetized on ice before dissection, and one biological replicate was prepared by pooling the hepatopancreas from one shrimp sampled from each of three parallel groups; three biological replicates were prepared for each group. For subsequent RNA extraction and transcriptomic analysis, all samples were snap-frozen in liquid nitrogen and maintained at −80 °C. All shrimp for sampling were anesthetized on ice before dissection. After completion of the experiment, all surviving shrimp and carcasses were treated as laboratory biological waste and disposed of hygienically by the institutional animal facility; none of the animals entered the human food chain, and culture water and equipment were disinfected before discharge, in accordance with national guidelines for laboratory animal welfare (GB/T 35892–2018) [70]. Figure 14 illustrates the experimental design, detailing the acclimation process, treatment groups, sampling time points, and the analysis methods employed in this study.

Determination of sulfate ions and major ions in water: The concentration of sulfate ions in water was determined using the sodium sulfate precipitation titration method with alizarin red as an indicator [71]. Ion chromatography was employed to determine Na^+^ and Cl^−^ concentrations in water (JY/T 0575-2020); acid–base titration was used to measure CO_3_^2−^ and HCO_3_^−^ concentrations in water (GB 8538-2022); and inductively coupled plasma emission spectroscopy (ICP) was applied to determine K^+^, Ca^2+^, and Mg^2+^ concentrations in water.

Acute toxicity test (LC_50_): The acute toxicity of sulfate to *E. carinicauda* was determined using 75 L white PVC cylindrical tanks. Based on preliminary trials, natural seawater was used as the control group (SO_4_^2−^ concentration of 30 mmol/L), and analytical-grade sodium sulfate (Na_2_SO_4_, Sinopharm Chemical Reagent Co., Ltd., Shanghai, China) was added to seawater to prepare solutions with SO_4_^2−^ concentrations of 64, 98, 132, 166, and 200 mmol/L. After being allowed to stabilize for 24 h, these solutions were used for the experiment. For each concentration, three replicate tanks were set up with 10 shrimp per tank, and the shrimp were exposed for 96 h. During the exposure period, one-third of the water in each tank was replaced daily with water of the corresponding sulfate concentration. The cumulative number of dead shrimp in each group was recorded at 0, 24, 48, 72, and 96 h, the mortality rate was calculated, and dead individuals were removed promptly. The median lethal concentration (LC_50_) of sulfate for *E. carinicauda* was calculated using probability analysis based on the modified Käber method in SPSS 27.0.

### 4.2. Observation of Tissue and Ultrastructural Changes

At 0, 24, and 48 h, live shrimp were randomly selected from each group of tanks, and hepatopancreas tissues were collected, fixed in 10% formaldehyde solution, embedded in paraffin, and stained with hematoxylin–eosin (H.E). Histological alterations were examined using optical microscopy. Hepatopancreatic and gill tissues were initially fixed in 2.5% glutaraldehyde, washed three times with phosphate-buffered saline (PBS), and subsequently post-fixed in 1% osmium tetroxide at 4 °C for 2 h. The tissues were subsequently rinsed three times with distilled deionized water (ddH_2_O) and dehydrated using an ethanol gradient (30%, 50%, 70%, 80%, 95%, 100%) for 15 min at each concentration. Samples were transitioned to propylene oxide and embedded in 812 resin via graded infiltration; polymerization was performed at 60 °C. Semi-thin and ultrathin sections were prepared using a Leica UC7 ultramicrotome (Leica Microsystems GmbH, Wetzlar, Germany), stained with uranyl acetate and lead citrate, and analyzed using transmission electron microscopy (TEM).

### 4.3. Transcriptomics Analysis

For each sample, total RNA was extracted from approximately 100 mg of hepatopancreas tissue using TRIzol reagent (Invitrogen, Carlsbad, CA, USA). The quality of the RNA was assessed through RNase-free agarose gel electrophoresis and an Agilent 2100 Bioanalyzer (Agilent Technologies, Palo Alto, CA, USA). mRNA was isolated using oligo(dT) beads (Epicentre, Madison, WI, USA) and fragmented into short segments. The fragments were reverse-transcribed into first-strand cDNA using random primers. RNase H was used to degrade the RNA strand in RNA–DNA hybrids, and second-strand cDNA was synthesized with DNA polymerase I in the presence of dNTPs and buffer. Double-stranded cDNA was purified using AMPure XP beads (Beckman Coulter, Beverly, MA, USA), PCR-amplified, and used to construct sequencing libraries, which were then sequenced on the Illumina HiSeq™ 6000 platform by Gene Denovo (Guangzhou, China). Raw reads were processed using fastp (version 0.18.0) [72] to remove reads containing synonymous or low-quality bases. These reads were aligned against an rRNA database using Bowtie2 (version 2.2.8) [73] to filter out rRNA-mapped reads. The remaining clean reads were aligned using HISAT2 (version 2.4) [74] with the *E. carinicauda* reference genome (ncbi_GCF_036898095.1). Mapped reads from each sample were assembled using StringTie (version 1.3.1) [75,76], and gene expression levels were quantified using FPKM via RSEM [77].

In the bioinformatics analysis phase, a principal component analysis (PCA) model was first constructed using the gmodels package to evaluate relationships among samples. Differential expression gene (DEG) screening employed the DESeq2 (v1.20.0) [78] software, setting (|log2FC| ≥ 1 and FDR < 0.05) as significance thresholds for DEGs. To gain deeper insights into the biological functions of these DEGs, their associated KEGG pathways were identified through the KEGG database.

### 4.4. Proteomic Analysis

Protein extraction, enzymatic digestion, and data acquisition were carried out according to the following steps. Protein extraction was performed by suspending samples in lysis buffer (1% SDS, 8M urea) with a 1× protease inhibitor cocktail. After vortexing and grinding twice with a high-throughput tissue grinder, the mixture was incubated at 4 °C for 30 min with vortexing every 10 min. Samples were centrifuged at 16,000× *g* for 20 min at 4 °C, and the supernatant protein concentration was determined using a BCA protein assay kit. For proteolysis, proteins were denatured, reduced, alkylated, and digested with trypsin using the iST Sample Preparation Kit. Briefly, 50 µL of lysis buffer was added and the mixture was heated at 95 °C for 10 min with agitation. After cooling, trypsin digestion buffer was added and samples were incubated at 37 °C for 2 h. Digestion was stopped with stop buffer, and peptides were cleaned and desalted using the iST cartridge, eluted, lyophilized by SpeedVac, and stored for LC–MS analysis. Data acquisition was carried out on an UltiMate 3000 LC system connected to the timsTOF Pro 2 mass spectrometer. Samples were separated on an AUR3-15075C18 column with a 60 min gradient, and data-independent acquisition (DIA) data was acquired in diaPASEF mode with 22 × 40 Th precursor isolation windows. The MS1 cycle time was adjusted with variable steps, and collision energy was ramped from 59 to 20 eV during PASEF MSMS scanning.

Mass spectrometry data were processed in Spectronaut (version 18; Biognosys AG, Schlieren, Switzerland) using a standardized analysis workflow. Carbamidomethylation of cysteine was specified as a fixed modification and methionine oxidation as a variable modification. Retention times were calibrated with the dynamic iRT algorithm. To control false positives, a target–decoy approach employing a mutated decoy database was applied, and false discovery rates (FDRs) at both the precursor and protein levels were constrained to <1%. For quantification, batch effects were corrected by locally weighted scatterplot smoothing (LOESS), and protein intensities were estimated with MaxLFQ from high-confidence peptides (q value < 0.01), ensuring accurate and reproducible protein-abundance measurements.

The assessment of inter-sample relationships was achieved through principal component analysis (PCA), performed using the gmodels package. Differentially expressed proteins (DEPs) were identified by Student’s *t*-test with Benjamini–Hochberg correction, using significance thresholds of (|log2FC|) ≥ 1 and FDR < 0.05. To further elucidate the biological functions of the DEPs, Kyoto Encyclopedia of Genes and Genomes (KEGG) pathway analysis was conducted to identify pathways associated with these proteins.

### 4.5. Validation of DEGs by RT-qPCR

To confirm the RNA-seq findings, the expression levels of nine randomly chosen differentially expressed genes (DEGs) were assessed by quantitative PCR (qPCR). The primers used for qPCR, along with their corresponding genes IDs and amplification efficiencies are listed in Table 1. The housekeeping gene 18S rRNA was used as an internal control. In summary, qPCR was performed using TB Green^®^ Premix Ex Taq™ II (TaKaRa Bio, Beijing, China) following the manufacturer’s instructions on a StepOnePlus™ Real-Time PCR System (Applied Biosystems, Foster City, CA, USA). Transcriptome samples from three biological replicates at each time point were used, with each biological sample analyzed in technical triplicates. The relative expression of target genes was calculated using the 2^−ΔΔCt^ method [79].

### 4.6. Statistical Analysis

Histomorphometric analyses were performed in Fiji/ImageJ by applying an identical thresholding workflow to images acquired at the same magnification, followed by particle analysis to quantify luminal (H&E) or vesicle/vacuole (TEM) area fraction and object counts. All experimental data, including histomorphometric readouts, were statistically analyzed using GraphPad Prism 8.0.2, Microsoft Excel 2015, and SPSS 27.0. Results are expressed as mean ± standard deviation (x^−^ ± SD). One-way analysis of variance (ANOVA) was performed in SPSS 27.0, and differences between means were evaluated using the LSD multiple comparison test. A significance level of *p* < 0.05 was adopted.

## 5. Conclusions

This study integrates histological, transcriptomic, and proteomic analyses to systematically elucidate the damage inflicted by sulfate-type saline–alkaline water on the hepatopancreas of *E. carinicauda* and the corresponding adaptive mechanisms. The results indicate that acute sulfate stress triggers energy-metabolic reprogramming in the hepatopancreas, with glycolysis being suppressed while oxidative phosphorylation is enhanced to maintain energetic homeostasis. Concurrently, key nodes of one-carbon metabolism (e.g., SHMT2) and enzymes associated with the glycolysis–serine shunt (e.g., PGAM2) secure the intracellular supply of reducing power and thereby mitigate oxidative injury. The stress further perturbs ion-transport systems, with components of Ca^2+^ signaling and the cAMP pathway (e.g., SLC8A1, ADCY9) being activated, coupling energy allocation to the regulation of intracellular ion balance. Collectively, these findings provide important insight into the stress-response mechanisms of *E. carinicauda* under acute sulfate challenge and furnish a theoretical basis and candidate molecular targets for selective breeding aimed at improved stress resistance. From an aquaculture perspective, our results thus provide mechanistic guidance for managing sulfate-type saline–alkaline waters (e.g., optimizing water quality and stocking conditions) and for designing breeding and husbandry strategies to enhance the robustness of *E. carinicauda* and other cultured crustaceans in sulfate-dominated environments. Nevertheless, the present multi-omics analysis is limited to a single acute exposure regime, and the key candidate regulators identified here have not yet been functionally validated or integrated with metabolomic profiles and long-term farming performance; addressing these limitations will be essential in future studies to fully translate our findings into practical applications for aquaculture.

## Figures and Tables

**Table 1:** Primer sequences used in this study.

Gene Name	NCBI Accession Number	Forward Primer (5′-3′)	Reverse Primer (5′-3′)	Amplification Efficiency
*18S rRNA*	GQ369794.1	GCTATGCGAGCAGGTTTC	CATGGCGTGACAGTTTCC	98.06%
*ADH5*	XM_068390262.1	GGTTCCACCTGTGCTGTC	TGCTCGTTCGGGTTGTAG	94.19%
*4cl3*	XM_068390317.1	GGAATGACGGAAGTGTTA	GTGAGTTGGAGGAAGGTC	108.53%
*TUBA1A*	XM_068346384.1	GCCGCAGGAACTTAGACAT	GGGCATAGGTAACGAGGG	100.72%
*Mtarc2*	XM_068390020.1	GGCGACTCCACATTACCT	GCTCGATCCGTCTTCTTAT	95.27%
*Bco1*	XM_068370955.1	ACGAATAAACCACCCGTCAA	CTCCGTTCCACTCAAATCCA	92.88%
*YMR099C*	XM_068383251.1	CGACGAGTGTTGTGGTTC	GGTATTCCTCCTCTTATGG	109.52%
*Arsb*	XM_068353702.1	GCAACAATGAAGACCCAG	AAAGCCAAATACAGGAACA	97.66%
*Slc31a1*	XM_068387454.1	TGGAGGGAAGACCTGATG	CGAACAAGAAGTAACCGAGT	95.32%
*Ctsc*	XM_068372694.1	ATACCTTGGGCGAGATTG	TCGTGAGACCACCCGTTC	98.18%

## Data Availability

Transcriptome data involved in this present study have been deposited in Sequence Read Archive (SRA) (https://www.ncbi.nlm.nih.gov/bioproject (accessed on 19 June 2025)), BioProject accession number PRJNA1279435 (SRA: SUB15400189). The mass spectrometry proteomics data have been deposited to the ProteomeXchange Consortium (https://www.iprox.cn/page/project.html (accessed on 23 December 2025)) via the iProX partner repository (ProteomeXchange identifier: PXD072331; iProX accession: IPX0014828000).

## Supplementary Materials

The following supporting information can be downloaded at: https://www.mdpi.com/article/10.3390/ijms27021056/s1.

## Abbreviations

H.E	hematoxylin–eosin
PBS	phosphate-buffered saline
ddH_2_O	distilled deionized water
TEM	transmission electron microscopy
ALU	abnormal lumen
Mv	microvilli
Nu	nucleolus
ER	endoplasmic reticulum
RNA-seq	RNA sequencing
PCR	polymerase chain reaction
DEG/DEGs	differentially expressed gene(s)
RT-qPCR	quantitative real-time PCR
DEP/DEPs	differentially expressed protein(s)
SDS	sodium dodecyl sulfate
GO	Gene Ontology
KEGG	Kyoto Encyclopedia of Genes and Genomes
LC_50_	median lethal concentration
OXPHOS	oxidative phosphorylation
ROS	reactive oxygen species
NAD^+^	nicotinamide adenine dinucleotide (oxidized form)
NADPH/GSH	reducing power: NADPH and glutathione
cAMP	cyclic adenosine monophosphate
NCX	Na^+^/Ca^2+^ exchanger
HK2	Hexokinase 2
ENO	Enolase
GAPDH	Glyceraldehyde-3-phosphate dehydrogenase
PKM	Pyruvate kinase, muscle
ADCY9	Adenylyl cyclase 9
PGAM2	Phosphoglycerate mutase 2
GPX	Glutathione peroxidase
CAT	Catalase
