# Supplementary material for: Multi-Omics Profiling of the Hepatopancreas of Ridgetail White Prawn Exopalaemon carinicauda Under Sulfate Stress
Source: Int J Mol Sci. 2026 Jan 21;27(2):1056. doi: 10.3390/ijms27021056 (PMC12842194; doi:10.3390/ijms27021056)
Supplement: Supplementary file 1 [file ijms-27-01056-s001.zip › Table S1.pdf]

Table S1 Changes in water pH and salinity at different sulfate concentrations

n=3; x±SD

Sulfate concentration /(mmol/L)	Na_2SO_4 addition /(g/L)	calculated sulfate concentration /(mmol/L)	measured sulfate concentration /(mmol/L)	pH	salinity
30	0	30	30	7.85 ± 0.02^a	25.00 ± 0.50^f
64	5	65.20	64	7.96 ± 0.03^a	27.94 ± 0.03^e
98	10	100.40	98	8.03 ± 0.09^a	31.15 ± 0.04^d
132	15	135.60	132	8.05 ± 0.02^a	33.92 ± 0.14^c
166	20	170.81	166	8.02 ± 0.06^a	36.42 ± 0.25^b
200	25	206.01	200	8.03 ± 0.06^a	39.21 ± 0.10^a

Note: The different letters in the upper right corner of each column indicate significant differences (P<0.05).
