# Supplementary material for: Multi-Omics Profiling of the Hepatopancreas of Ridgetail White Prawn Exopalaemon carinicauda Under Sulfate Stress
Source: Int J Mol Sci. 2026 Jan 21;27(2):1056. doi: 10.3390/ijms27021056 (PMC12842194; doi:10.3390/ijms27021056)
Supplement: Supplementary file 1 [file ijms-27-01056-s001.zip › Table S2.pdf]

**Table S2 Changes in the concentration of major ions in waters with different sulfate concentrations**

n=3; x±SD

| Sulfate concentration /(mmol/L) | Ion mass concentration /(mg/L) | | | | | | |
|---|---|---|---|---|---|---|---|
| | $K^+$ | $Na^+$ | $Ca^{2+}$ | $Mg^{2+}$ | $Cl^-$ | $CO_3^{2-}$ | $HCO_3^-$ |
| 30 ( pure seawater) | $296.00 \pm 8.49^a$ | $8063.73 \pm 107.03^c$ | $348.50 \pm 12.02^a$ | $979.50 \pm 6.36^a$ | $14138.39 \pm 55.96^a$ | $13.76 \pm 2.98^a$ | $145.18 \pm 3.03^a$ |
| 106 | $298.78 \pm 6.46^a$ | $12191.53 \pm 87.19^b$ | $349.76 \pm 10.52^a$ | $987.58 \pm 6.16^a$ | $14109.34 \pm 55.34^a$ | $16.34 \pm 2.41^a$ | $146.50 \pm 3.14^a$ |
| 200 | $301.05 \pm 7.19^a$ | $16233.03 \pm 98.37^a$ | $351.01 \pm 11.17^a$ | $991.50 \pm 6.74^a$ | $14080.90 \pm 53.19^a$ | $16.34 \pm 2.73^a$ | $147.80 \pm 2.79^a$ |

Note: The different letters in the upper right corner of each column indicate significant differences($P<0.05$).
