# Supplementary material for: Multi-Omics Profiling of the Hepatopancreas of Ridgetail White Prawn Exopalaemon carinicauda Under Sulfate Stress
Source: Int J Mol Sci. 2026 Jan 21;27(2):1056. doi: 10.3390/ijms27021056 (PMC12842194; doi:10.3390/ijms27021056)
Supplement: Supplementary file 1 [file ijms-27-01056-s001.zip › Table S7.pdf]

Table S7 Changes in water Temp, OD and pH under sulfate stress

n=3; x±SD

Time/h	Temp/℃	OD/mg/L	pH
0	24±0.5[a]	8.53±0.09[a]	7.85±0.02[a]
24	24±0.5[a]	8.47±0.07[a]	7.91±0.09[a]
48	24±0.5[a]	8.41±0.06[a]	7.93±0.03[a]

Note: The different letters in the upper right corner of each column indicate significant differences (P<0.05).
